# Physical Activity Enhancement to a Behavioral Weight Loss Program for Severely Obese Individuals: A Preliminary Investigation

**DOI:** 10.5402/2012/465158

**Published:** 2012-09-13

**Authors:** Jessica L. Unick, Kevin C. O'Leary, Dale S. Bond, Rena R. Wing

**Affiliations:** Weight Control and Diabetes Research Center, The Miriam Hospital and Brown Medical School, 196 Richmond Street, Providence, RI 02903, USA

## Abstract

Severe obesity is characterized by low physical activity (PA) and interventions to enhance PA are needed. Participants (45.0 ± 3.9 kg/m^2^) were randomized to a 6-month standard behavioral weight loss program (SBWL; *n* = 14) or SBWL+technology (SBWL+TECH; *n* = 15). Both groups received identical SBWL treatment and SBWL+TECH also received a wearable PA monitor, providing “real-time” feedback, and website access to monitor energy balance. 6-month retention was similar between groups (SBWL: 12/13 versus SBWL+TECH: 11/14 completers; *P* = 0.19) and adherence to wearing the armband was excellent (91.3% of days). Although differences in PA between groups did not meet conventional thresholds of significance, SBWL+TECH increased their moderate-to-vigorous intensity PA by 132.9 ± 216.8 min/week, which was 3 times greater than SBWL (44.8 ± 124.3 min/week; *P* = 0.27; Cohen's *d* = 0.50). There was a trend for SBWL+TECH to self-monitor for a greater proportion of days compared to SBWL (86.2 ± 21.4% versus 71.5 ± 19.4%; *P* = 0.098; Cohen's *d* = 0.72). The difference in weight loss between groups was modest (SBWL+TECH: −10.0 ± 7.1% versus SBWL: −7.8 ± 6.7%; *P* = 0.46). These preliminary findings suggest that PA monitors may be one strategy for increasing PA among the severely obese. Larger, long-term trials are needed.

## 1. Introduction

Severe obesity (BMI ≥ 40 kg/m^2^), a rapidly rising subgroup of the obese population, is associated with increased risk for cardiovascular disease (CVD) and all-cause mortality [1, 2]. While the majority of clinical trials have focused on examining the efficacy of bariatric surgery, pharmacotherapy, or residential treatment camps, recently there has been a renewed interest in standard behavioral weight loss (SBWL) programs for treating the severely obese [3].

 Two recent studies suggest that SBWL programs for the severely obese produce significant 1-year weight losses and improvements in CVD risk factors, similar to their less obese peers [4, 5]. However, individuals with severe obesity report less physical activity (PA) both before and after the intervention [5]. This is of concern given the importance of PA on health outcomes [6, 7] and weight maintenance [8, 9]. Therefore, it is necessary to develop methods for increasing PA in this population, which if sustained over time, could favorably impact body weight.

One strategy for improving PA within SBWL treatment is through the use of wearable PA monitors. These monitors provide “real-time” PA and energy-expenditure feedback and are believed to improve energy balance awareness and reduce the burden of self-monitoring, thereby enhancing motivation and program adherence. Adding this technology to SBWL treatment (SBWL+TECH) for overweight or mildly obese individuals resulted in greater improvements in self-monitoring, self-reported PA, and weight loss at 6 months compared to SBWL alone [10]. Given their low PA, it seems appropriate to test the efficacy of this technology in the severely obese [5, 11]. This study examined whether adding wearable PA monitors to SBWL treatment for severely obese individuals improved PA and self-monitoring following a 6-month intervention. Although we also examined changes in body weight, based upon previous literature we hypothesized that the addition of technology would only have a modest effect on weight loss.

## 2. Methods and Procedures

### 2.1. Participants

 Twenty-nine individuals were randomized to SBWL (*n* = 14) or SBWL+TECH (*n* = 15). Participants were 21–55 years old, had a BMI ≥ 40 kg/m^2^, and no history of diabetes or bariatric surgery. Moreover, participants reported exercising <150 min/wk at baseline, had no medical contraindications to PA, regular computer and internet access, and passed a behavioral interview prior to randomization.

### 2.2. Procedures

At baseline and 6 months, body weight was measured and PA was objectively assessed for 1-week using the Sensewear armband (Body Media, Pittsburgh, PA, USA). Total minutes/week spent in moderate-to-vigorous intensity physical activity (MVPA) was computed. At 6-months, SBWL+TECH completed a questionnaire assessing the acceptability of the technology. All study procedures were approved by the Miriam Hospital's institutional review board.

### 2.3. Standard Behavioral Weight Loss Program

 The 6-month SBWL program consisted of weekly group meetings focusing on behavioral approaches to PA and dietary change. A structured exercise goal progressing to 250 min/week and a calorie intake goal of 1500–1800 kcal/day were prescribed to produce a 1-2 lb/week weight loss. Participants were instructed to monitor food intake and PA behaviors daily using paper diaries, which were returned with written feedback weekly.

### 2.4. Standard Behavioral Weight Loss Program Plus Technology Component

 SBWL+TECH received the SBWL program described above. In addition, participants were instructed to use the Body Media FIT system (Body Media Inc, Pittsburgh, PA) daily, consisting of an armband, digital display watch, and access to a website where food intake, body weight, and PA are monitored. The armband, which is worn on the upper arm, objectively assesses PA and energy expenditure and provides up-to-the-minute feedback (e.g., total steps, total MVPA minutes, total energy expenditure, and progress towards goals) via digital display. Participants were instructed to upload armband data to the website daily, which was accessed by the interventionist weekly and written feedback was provided.

### 2.5. Statistical Analyses

 Independent *t*-tests and chi-square analyses were used to compare treatment groups on demographic, baseline, and adherence variables. One-way ANOVA's were performed to compare SBWL and SBWL+TECH on changes in body weight and MVPA at 6 months, controlling for age, PA monitor wear time, and baseline variables where appropriate. Both completer's analyses and intent-to-treat (ITT) analyses (e.g., baseline weight carried forward) were performed when assessing changes in weight and PA. Effect sizes were calculated using Cohen's *d*. All analyses were conducted using SPSS for Windows (Version 18, Chicago, IL, USA).

## 3. Results

### 3.1. Participants

 Across treatment arms, the mean BMI (45.0 ± 3.9 kg/m^2^), body weight (126.8 ± 18.0 kg), and demographic characteristics of study participants (82% female, 79% Caucasian) were similar (*P* > 0.05). However, SBWL participants were significantly older than SBWL+TECH (46.1 ± 9.1 versus 38.7 ± 9.3 years; *P* = 0.04). Six-month retention did not differ between groups (SBWL: 12/13 versus SBWL+TECH: 11/14 completers; *P* = 0.19). Those who are not completing the intervention were significantly younger compared to completers (33.6 ± 10.1 versus 44.1 ± 8.4 years; *P* = 0.03); however groups were similar on all other measures (*P* > 0.40).

### 3.2. Adherence

 The proportion of treatment sessions attended did not differ between groups (SBWL: 75.8 ± 19.6% versus SBWL+TECH: 74.6 ± 26.6%; *P* = 0.90). Among completers, self-monitoring of dietary intake was considerably greater in SBWL+TECH compared to SBWL (86.2 ± 21.4% versus 71.5 ± 19.4% of possible days; Cohen's *d* = 0.72), although this did not reach conventional levels of significance (*P* = 0.098). Moreover, SBWL+TECH wore the armband for 91.3% of possible days for an average of 14.4 hours/day.

### 3.3. Feasibility of Technology Component

One-hundred percent of participants completing the study indicated that the technology improved the ease of self-monitoring and increased their motivation to adhere to exercise and weight loss goals. Moreover, all but 1 participant reported a desire to use the FIT system in the future to monitor exercise and weight loss progress, with 90% of those reporting they would wear the armband daily.

### 3.4. Changes in Physical Activity and Body Weight

**Figure 1:**
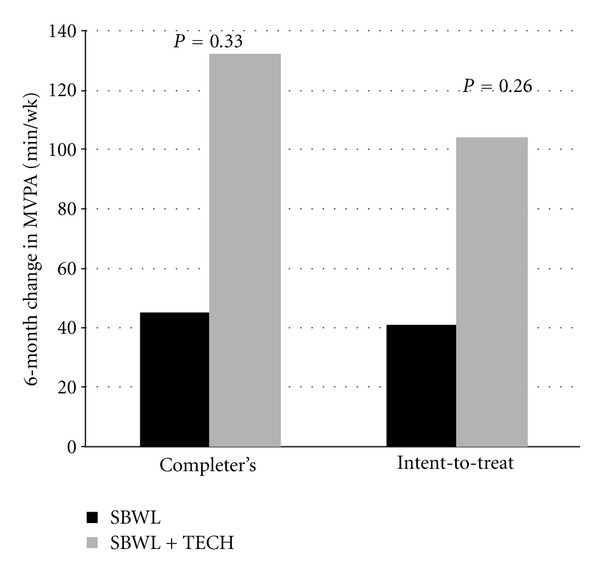
Baseline to 6-month changes in physical activity stratified by treatment arm and type of analyses performed. MVPA = moderate-to-vigorous intensity physical activity; SBWL = standard behavioral weight loss intervention; SBWL + TECH = standard behavioral weight loss program plus technology component.

Both intervention arms significantly increased MVPA from baseline to 6 months (*P* < 0.05). Although not statistically significant (*P* = 0.33), completer's analyses revealed that the increase in MVPA was approximately 3 times greater in SBWL+TECH (133.0 ± 217 min/wk) compared to SBWL (44.8 ± 124.6 min/wk), which was a moderate effect (Cohen's *d* = 0.50; Figure 1). Both treatment arms lost a significant amount of weight at 6 months (*P* < 0.001); however, percent weight loss did not differ between groups for either completer's analyses (SBWL: 7.8 ± 6.7 versus SBWL+TECH: 10.0 ± 7.1%; *P* = 0.46; Cohen's *d* = 0.32) or intent-to-treat analyses (SBWL: 7.2 ± 6.8 versus SBWL+TECH: 7.9 ± 7.6%; *P* = 0.83).

## 4. Discussion

 This study was the first to examine the feasibility of adding an objective PA monitor to SBWL treatment for severely obese individuals. Furthermore, it was the first to report objectively-assessed changes in PA following an SBWL intervention. This is significant given the low levels of PA and previously observed discrepancy between self-report and objective MVPA in this population [12]. In the current study, 6-month changes in objectively measured MVPA were low in SBWL (45 min/week) and three times as large in SBWL+TECH (133 min/week). Although not statistically significant, the 0.50 effect size observed is relatively high for PA intervention trials [13]. Moreover, these objective findings are in concordance with previous studies in nonseverely obese cohorts, which reported that the change in self-report MVPA was 2 [10] and 4 times [14] greater in SBWL+TECH compared to SBWL. Given the low PA levels among the severely obese [5, 11], and the associated health ramifications [6, 7], adding PA monitors to SBWL may be one strategy for increasing PA.

 It is well documented that the effect of PA on initial weight loss is modest (~2-3 kg) [15], thus the additional 2.2% weight loss achieved by SBWL+TECH is not surprising. This magnitude of difference in weight loss between groups may be associated with the additional 88 min/wk (~400–500 kcal/wk) increase in PA observed in SBWL+TECH. Moreover, this finding is in accordance with previous studies utilizing this technology in less obese individuals which reported an additional 2% weight loss in SBWL+TECH compared to SBWL [10, 14]. Given that high PA is a primary predictor of weight maintenance [8], it is plausible that similar technology-based approaches may be efficacious for increasing PA and improving long-term weight loss success. Future trials should examine this question.

This preliminary study also demonstrates the feasibility of adding objective PA monitors to SBWL treatment for the severely obese. On average, SBWL+TECH wore the armband for >90% of possible days and 90% of waking hours, with 100% of participants reporting that the technology increased motivation and improved the ease of self-monitoring. Moreover, SBWL+TECH self-monitored for an additional 15% of possible days, compared to SBWL, which is considered a moderate-to-large effect size, although not statistically significant. Compliance to using this technology in the long term warrants further investigation.

This study is strengthened by the use of objective measures to capture 6-month changes in PA, and also through the application of this technology to a severely obese cohort. However, this pilot study was not powered to detect significant differences in PA between treatment arms. It is estimated that 128 individuals (*n* = 64/group) are needed to detect the observed effect size of 0.50 with 80% power at *P* < 0.05. Additionally, it is unclear whether the acceptability ratings of the TECH system were similar in completers and noncompleters. However, of the 3 SBWL+TECH noncompleters, none stated dropping out due to a disliking of the technology.

These findings add to the scant literature demonstrating that severely obese individuals can achieve significant weight losses through SBWL treatment and that adding technology with real-time PA feedback is well accepted and may increase self-monitoring and PA in this population. Additional research involving larger samples is needed to determine whether adding this technology can enhance the efficacy of SBWL programs in the long term.

## References

[1] Flegal KM, Carroll MD, Kit BK, Ogden CL (2012). Prevalence of obesity and trends in the distribution of body mass index among US adults, 1999-2010. *Journal of the American Medical*.

[2] McTigue K, Larson JC, Valoski A (2006). Mortality and cardiac and vascular outcomes in extremely obese women. *Journal of the American Medical Association*.

[3] Blackburn GL, Wollner S, Heymsfield SB (2010). Lifestyle interventions for the treatment of class III obesity: a primary target for nutrition medicine in the obesity epidemic. *American Journal of Clinical Nutrition*.

[4] Goodpaster BH, DeLany JP, Otto AD (2010). Effects of diet and physical activity interventions on weight loss and cardiometabolic risk factors in severely obese adults: a randomized trial. *Journal of the American Medical Association*.

[5] Unick JL, Beavers D, Jakicic JM, Kitabchi AE, Knowler WC, Wadden TA (2011). Effectiveness of lifestyle interventions for individuals with severe obesity and type 2 diabetes: results from the Look AHEAD trial. *Diabetes Care*.

[6] Wei M, Kampert JB, Barlow CE (1999). Relationship between low cardiorespiratory fitness and mortality in normal-weight, overweight, and obese men. *Journal of the American Medical Association*.

[7] Fogelholm M (2010). Physical activity, fitness and fatness: relations to mortality, morbidity and disease risk factors. A systematic review. *Obesity Reviews*.

[8] Unick JL, Jakicic JM, Marcus BH (2010). Contribution of behavior intervention components to 24-month weight loss. *Medicine and Science in Sports and Exercise*.

[9] Klem ML, Wing RR, McGuire MT, Seagle HM, Hill JO (1997). A descriptive study of individuals successful at long-term maintenance of substantial weight loss. *American Journal of Clinical Nutrition*.

[10] Pellegrini CA, Verba SD, Otto AD, Helsel DL, Davis KK, Jakicic JM (2012). The comparison of a technology-based system and an in-person behavioral weight loss intervention. *Obesity*.

[11] Bond DS, Jakicic JM, Vithiananthan S (2010). Objective quantification of physical activity in bariatric surgery candidates and normal-weight controls. *Surgery for Obesity and Related Diseases*.

[12] Bond DS, Jakicic JM, Unick JL (2010). Pre- to postoperative physical activity changes in bariatric surgery patients: self report vs. objective measures. *Obesity*.

[13] Holtzman J, Schmitz K, babes G, Kane RL, Duval S, Wilt TJ (2004). Effectiveness of behavioral interventions to modify physical activity behaviors in general populations and cancer patients and survivors. *Evidence Report/Technology Assessment*.

[14] Polzien KM, Jakicic JM, Tate DF, Otto AD (2007). The efficacy of a technology-based system in a short-term behavioral weight loss intervention. *Obesity*.

[15] Donnelly JE, Blair SN, Jakicic JM, Manore MM, Rankin JW, Smith BK (2009). Appropriate physical activity intervention strategies for weight loss and prevention of weight regain for adults. *Medicine and Science in Sports and Exercise*.

